# SPA-Net: A Deep Learning Approach Enhanced Using a Span-Partial Structure and Attention Mechanism for Image Copy-Move Forgery Detection

**DOI:** 10.3390/s23146430

**Published:** 2023-07-15

**Authors:** Kaiqi Zhao, Xiaochen Yuan, Zhiyao Xie, Yan Xiang, Guoheng Huang, Li Feng

**Affiliations:** 1 School of Computer Science and Engineering, Macau University of Science and Technology, Macao 999078, China; 2009853dii30002@student.must.edu.mo (K.Z.); 2009853gia30006@student.must.edu.mo (Y.X.); lfeng@must.edu.mo (L.F.); 2 Faculty of Applied Sciences, Macao Polytechnic University, Macao 999078, China; p2215884@mpu.edu.mo; 3 School of Computer Science and Technology, Guangdong University of Technology, Guangzhou 510006, China; kevinwong@gdut.edu.cn

**Keywords:** copy-move forgery detection, cross partial structure, feature extraction, attention mechanisms

## Abstract

With the wide application of visual sensors and development of digital image processing technology, image copy-move forgery detection (CMFD) has become more and more prevalent. Copy-move forgery is copying one or several areas of an image and pasting them into another part of the same image, and CMFD is an efficient means to expose this. There are improper uses of forged images in industry, the military, and daily life. In this paper, we present an efficient end-to-end deep learning approach for CMFD, using a span-partial structure and attention mechanism (SPA-Net). The SPA-Net extracts feature roughly using a pre-processing module and finely extracts deep feature maps using the span-partial structure and attention mechanism as a SPA-net feature extractor module. The span-partial structure is designed to reduce the redundant feature information, while the attention mechanism in the span-partial structure has the advantage of focusing on the tamper region and suppressing the original semantic information. To explore the correlation between high-dimension feature points, a deep feature matching module assists SPA-Net to locate the copy-move areas by computing the similarity of the feature map. A feature upsampling module is employed to upsample the features to their original size and produce a copy-move mask. Furthermore, the training strategy of SPA-Net without pretrained weights has a balance between copy-move and semantic features, and then the module can capture more features of copy-move forgery areas and reduce the confusion from semantic objects. In the experiment, we do not use pretrained weights or models from existing networks such as VGG16, which would bring the limitation of the network paying more attention to objects other than copy-move areas.To deal with this problem, we generated a SPANet-CMFD dataset by applying various processes to the benchmark images from SUN and COCO datasets, and we used existing copy-move forgery datasets, CMH, MICC-F220, MICC-F600, GRIP, Coverage, and parts of USCISI-CMFD, together with our generated SPANet-CMFD dataset, as the training set to train our model. In addition, the SPANet-CMFD dataset could play a big part in forgery detection, such as deepfakes. We employed the CASIA and CoMoFoD datasets as testing datasets to verify the performance of our proposed method. The *Precision*, *Recall*, and *F1* are calculated to evaluate the CMFD results. Comparison results showed that our model achieved a satisfactory performance on both testing datasets and performed better than the existing methods.

## 1. Introduction

**Figure 1:**
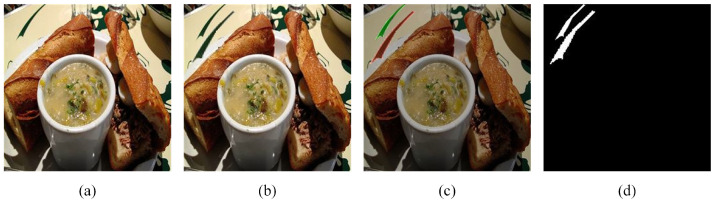
An example of image copy-move forgery in daily life: (**a**) is the original image, (**b**) is the copy-move image, (**c**) shows the copied and pasted regions, which are marked green and red, respectively, and (**d**) is the binary mask of the copy-move image.

**Figure 2:**
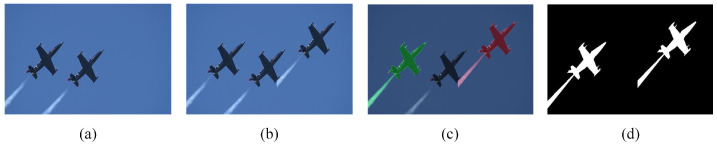
An example of image copy-move forgery in the military: (**a**) is the original image, (**b**) is the copy-move image, (**c**) shows the copied and pasted regions, which are marked green and red, respectively, and (**d**) is the binary mask of copy-move image.

With the development of computer science and technology, image forgery problems are increasingly emerging. Among them, image copy-move forgery detection has become a hot research direction. Image forensics can be divided into active and passive forensics. Image active forensic technology involves pre-inserting relevant information, which can be used to verify copyright ownership or detect tampering, such as watermarks and digital signatures. However, there are restrictions to both digital watermarking and signatures, because only images with a watermark or signature are detectable using these techniques. The passive image forensic techniques are of broader use, and copy-move forgery detection (CMFD) is one of the approaches that can deal with the problem of copied pieces coming from the same image, which is shown in Figure 1 and Figure 2.

Compared to copy-move forgery, splicing forgery is copying and pasting between different images. The tampered images have a lot of clues from edge artifacts, statistical features, and imaging features; for example, a color filter array (CFA) consists of a series of color sensors, CFA interpolation reconstructs a full color image by converting the captured output into red, green, and blue primary color channels (red, green, and blue, RGB), and different visual sensors have different interpolation algorithms. In [1], image splicing was detected using the difference of sensor noise power in CFA interpolated pixels, when copy-move forgery occurred within the same image.The statistical properties of a real region and a tampered region can be very similar, so some splicing forgery detection methods [2,3,4,5] do not have advantages for CMFD. Therefore, various methods have been presented for detecting copy-move forgery, whose main steps are feature extraction and feature matching.

Traditional CMFD includes block-based approaches and keypoint-based approaches. The primary idea of block-based approaches is to divide an image into a number of patches, and to compare these with each other to find the matched regions. Kakar and Sudha [6] proposes to extract blur moment invariant features as block features and then employ a polar cosine transform (PCT) to match block features. Singular value decomposition (SVD) is designed to extract features for each small overlapping block, and a discrete cosine transform (DCT) is applied for capturing discriminating features. Various other papers also proposed different ideas: in [7], blur moment in-variants are used to better detect the blur region, and principal component analysis (PCA) is adopted to reduce the dimensions, as well as to find features that are easier to understand by taking the greatest individual differences, which are shown by the main components. However, the disadvantage is that the computational cost is very high. Next, ref. [8] proposes using singular value decomposition (SVD) to reduce the feature description and decrease the computational cost, and the morphological opening refines the result, to avoid further losses. However, the robustness against geometric transformations was limited. After extracting the features, different feature matching methods have been proposed. In [8], a K-dimensional tree (KD-Tree) is used to achieve matching. In the feature map that is divided into blocks, each block could find the one with the highest similarity. The similarity standard is determined using a given threshold, whereas only those whose similarity was equal to or greater than the threshold could be recorded. Next, the similar blocks are eliminated if their neighbors were different. In [9], an approximate nearest neighbor search is performed by hashing vectors using a set of hash functions, which is designed to obtain values that had the same hash. Locality-sensitive hashing (LSH) guarantees that a given vector and its approximate nearest neighbor can be mapped to the hash with a high probability.

Keypoint-based methods are also essential types of CMFD. The core of key-point-based methods is to extract distinctive local features, such as corners, blobs, and edges from an image. Each feature is presented with a set of descriptors produced within a region around the features. The descriptor is used to increase the reliability of the features for the affine transformation. Scale-invariant feature transform (SIFT) and speeded up robust features (SURF) have been widely used to extract key points in images. SIFT techniques are highly robust against post processing and inter-mediate operations; however, they are computationally complex and incapable of determining forgeries in areas which are flat, due to a lack of reliable key points. SURF features have been proposed to improve the performance of SIFT. SIFT [10] has become indispensable in this area. Ref. [11] takes advantage of SIFT and best-bin-first. Then, refs. [12,13,14] proposed an improvement on this basis. SIFT has driven the development of SURF [15]. The two methods have similar procedures: local feature point extraction, feature point description, and feature point matching. It is worth mentioning that SURF has two tools to increase the execution efficiency, one is the use of an integral graph in Hessian (Hesse matrix), and the other is the use of a dimension reduction feature descriptor. The Harris corner detector extracts edges and corners from a region, and it was employed for keypoint extraction in [16]. In feature matching of a keypoint-based area, the nearest neighbor determines the similarity between points by calculating the distance between each point in the vector space. If the distance meets the specified threshold, the points are considered similar, which was applied in [11].

Although a lot of block-based and keypoint-based approaches have been proposed for CMFD, several flaws exist. Block-based techniques have high processing costs and undetectable large-scale distortion. Meanwhile, the performance in addressing smoothing forgery snippets is not good with keypoint-based techniques. Likewise, fusion methods integrate feature extraction from keypoint-based and region matting from block-based methods. Unfortunately, the smoothing forgery fragment is not addressed by the fusion method using sparse key point features. Auxiliary deep neural networks (DNNs) have recently been proposed, to help with various aspects of CMFD [17,18,19,20,21]. CMFD using DNN algorithms involves these basic steps: (1) a pre-processing stage, which involves transforming the image into the format that the network requires, such as scaling, data augmentation, format conversion, and so on; (2) a feature extraction stage, which is the process of extracting the image’s significant hierarchical characteristics, to be used in the following procedures; (3) feature matching stage, which includes block-based and key-point-based matching methods, and which is used to match the extracted features; and (4) the post-processing stage, which involves transforming the results into the format required, such as visualizing them in an image format. [17] proposed a deep learning technology-based method for detection of both splicing and copy-move forgery. This method contained 10 convolution layers, among which, the first layer was a spatial rich model (SRM) [22] pre-processing layer for feature extraction. [18] proposed using VGG16 for feature extraction and using self-correlation as a matching method. In [20], a pyramid feature extraction method was proposed. Reference [23] also applied an encoder-to-decoder network and used a classification layer to obtain the copy-move area. However, reference [17] could only identify whether the image had been tampered and could not locate the tampered area in the image. Meanwhile, reference [18] only performed matching in a pretrained VGG16, which put particular emphasis on the object instead of the forgery area. VGG16 is a model for image classification or target recognition, which contains rich semantic information. However, in CMFD, the tampered objects are not necessarily objects with rich semantic information but may be ordinary meaningless areas. Reference [20] showed this may lead to much useless information passing across the network model.The proposed SPA-Net is an end-to-end DNN method for solving the shortcomings of the existing deep learning methods, and our contributions can be listed as follows:

1 In the SPA-Net feature extractor, we use a combination of a span-partial structure and residual attention mechanism, to reduce the repeated gradient information and pay more attention to the copy-move forgery area. To the best of our knowledge, this structure has not been used in the previous CMFD methods;
2 We use a feature matching module to locate the copy-move regions by calculating the similarity between features. To avoid only locating the objects that have rich semantic information but not the copy-move areas, we use no pretrained models or weights;
3 Moreover, we generated a SPANet-CMFD dataset with original images from SUN [24] and COCO [25], and we selected the source area using Labelme manually in each image. In addition, to enhance the ability of SPA-Net, we not only selected the objects, but also the areas that contained less semantic information as copy-move regions.

This paper is organized as follows: Section 2 gives related studies on residual structure and attention mechanism; Section 3 describes the proposed SPA-Net for CMFD in detail; Section 4 shows the experimental results, where the performance of the proposed SPA-Net was measured, the comparisons with the state-of-the-art approaches were conducted, and the challenges and weaknesses that require further improvement are discussed; Finally, we give the conclusions and future work in Section 5.

## 2. Related Work

### 2.1. Residual Structure

To optimize the repeated gradient information in their network, Reference [26] proposed a cross-stage partial network (CSPNet), which divided the underlying feature graph into two parts (the same feature graph was fed into two different convolutions, and the number of channels output from the two convolution was halved) and then fused them through the proposed cross-stage hierarchical results. The main concept is to truncate the gradient flow and add a transition layer after truncation, to make the gradient flow propagate through different network paths and prevent different layers from learning repeated gradient information. Using CSPNet as a backbone can effectively enhance CNN’s learning ability and reduce the amount of calculation. In addition, CSPNet is easy to implement and generic enough to handle different architectures.Therefore, in SPA-Net, we propose a span-partial structure in SPA-Net feature extractor module, and the main concept is to truncate the gradient flow and add a transition layer after truncation to make the gradient flow propagate through different network paths and prevent different layers from learning repeated gradient information.

### 2.2. Attention Mechanism

In the area of deep learning, models often need to receive and process large amounts of data, but sometimes the equivalent processing of images is not necessary, because only a small part of certain data is important. To reduce this information redundancy and make areas of interest more prominent, reference [22] proposed a squeeze and excitation net (SENet) [27] as an improvement. The principle of SENet is to enhance the important features and weaken the unimportant features by controlling the weight. In addition, the flexibility of the SE module lies in the fact that it can be directly applied to the existing network architecture, such as [28], a mobile inverted bottleneck convolution (MBConv), whose core idea is to learn feature weights through the network. In the proposed SPA-Net feature extractor module, we use a combination of a span-partial structure and residual attention mechanism to reduce repeated gradient information and pay more attention to the copy-move forgery area that we are interested in. To the best of our knowledge, this structure has not been used in the previous CMFD methods. The principle of the residual attention mechanism is to enhance the important features and weaken the unimportant features, by controlling the weight, so as to make the extracted features more directional.

## 3. Proposed SPA-Net for CMFD

The aim of CMFD is to find the copy-move forgery area of images; however, most existing networks do not follow the core process of CMFD, some are biased toward image segmentation, while others are biased toward image target recognition. Although some methods [19,29] have been proposed using existing pretrained network models and parameters, these cannot achieve satisfactory results. Therefore, instead of using pretrained models, we propose a deep learning approach without any pretrained models, and which uses a span-partial structure and attention mechanism (SPA-Net) for image CMFD. The SPA-Net is constructed using a sequence of DNN layers, and thus it can be trained from end to end.

**Figure 3:**
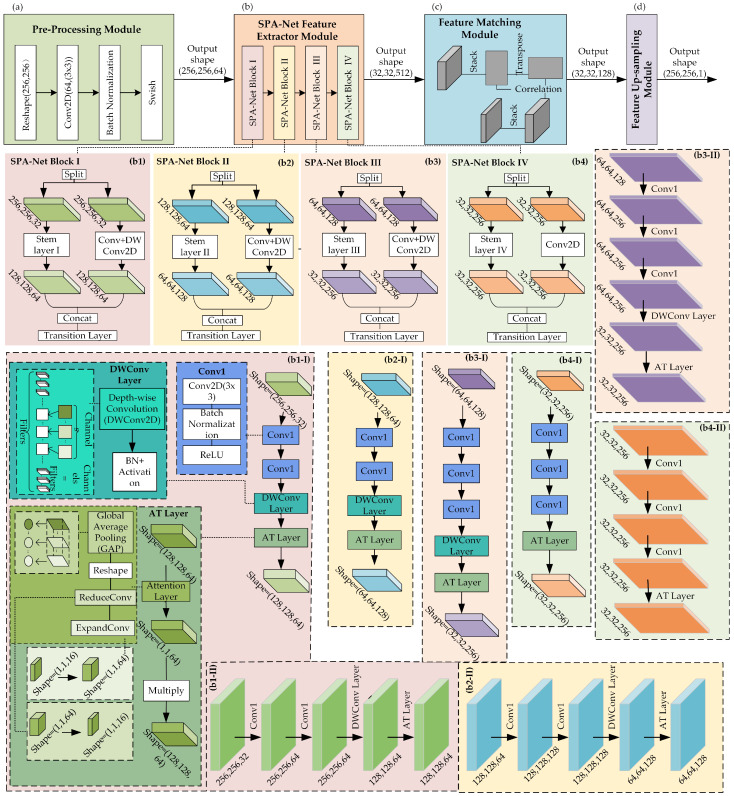
Overview of the proposed SPA-Net for CMFD. (**a**–**d**) denote the four modules of SPA-Net, (**b1**–**b4**) are the steps of four SPA-Net blocks, (**b1-I**–**b4-I**) are the details of each Stem Layer, (**b1-II**–**b4-II**) represent the shapes of intermediate outputs in Stem Layer I–IV.

Figure 3 shows an overview of the proposed SPA-Net for CMFD, which is composed of (a) preprocessing module, (b) SPA-Net feature extractor module, (c) feature matching module, and (d) feature upsampling module. The preprocessing module is used to process an input image of any size to the fixed size of 256×256×3 that is required by the network and to extract a rough feature map. The SPA-Net feature extractor module is composed of three blocks: SPA-Net Block I, SPA-Net Block II, and SPA-Net Block III. Each of the blocks includes two branch structures, where one is a stem layer that contains multiple residual structures and a convolution layer, and the other carries out convolution. The addition of a residual structure can increase the gradient value of backpropagation between layers and avoid the disappearance of the gradient caused by deepening, so that more fine-grained features can be extracted without worrying about network degradation. The feature matching module is used to learn the correlation between the features obtained with the SPA-Net feature extractor module. Finally, the feature upsampling module is used to resize the image to its original size.

### 3.1. Preprocessing Module

Before applying the SPA-Net feature extractor module, the preprocessing module as shown in Figure 3a is applied to adjust the input image to the format required by the model. During preprocessing, we first adjust the shape of the input image to fit the 256×256×3 required by our network and employ a Conv2D layer to reshape the image to extract a feature of size 256×256×64. Then, batch normalization is used to pull the layer eigenvalue distribution back to the standard normal distribution, and the eigenvalue falls in the interval where the activation function is sensitive to the input. Small changes in the input can lead to large changes in the loss function, making the gradient larger, avoiding the disappearance of the gradient, and speeding up the convergence. Finally, the swish activation function is applied to help prevent gradients from gradually approaching 0 and causing saturation during slow training. In this way, the preprocessing helps to speed up the processing of the image.

### 3.2. SPA-Net Feature Extractor Module

The SPA-Net feature extraction module aims to obtain a feature map with rich gradient information. The proposed SPA-Net feature extractor module is composed of four blocks: SPA-Net Block I, SPA-Net Block II, SPA-Net Block III, and SPA-Net Block IV, as shown in Figure 3(b1–b4). Each of the SPA-Net blocks includes dual span-partial structures, with the stem layer on the left branch and the layer of Conv_DWConv2D (CDW) on the right branch. A concatenate layer is used to combine the two branches in the end, and a traditional layer that contains convolution layer, BN, and activation function is used to further extract the feature map. The output of each stem layer and its fusion operations on the left and right branches can be defined as

(1) $$P_{i}=Transition\left(Concat\left[S_{i},C_{i}\right]\right),i\in \left\{1,\ldots ,4\right\}$$

where $P_{i}$ means the output of the corresponding SPA-Net block. Meanwhile, the subscript *i* also refers to the variables $S_{i}$ and $C_{i}$ used in the relevant SPA-Net block guidance by Equation (1), respectively.

Due to the feature map being of the same size, each SPA-Net Block uses a concatenate layer at the end, for the purpose of merging the feature maps from the left and right channel dimensions. The 256×256×64 feature map from the preprocessing layer is split into two feature maps, each with a size 256×256×32. One feature map uses stem layer I, while the other takes CDW to extract the features of interest, each with a size of 128×128×64, which are then connected together using concatenation. Using the split and merge strategy across stages can effectively reduce the possibility of duplication during the process of information integration and thus greatly improve the learning ability of the network.One branch stem layer contains multiple residual structures and convolution layers and then can increase the gradient value of backpropagation between layers and avoid the disappearance of the gradient caused by deepening, so that more fine-grained features can be extracted, without worrying about network degradation. Another branch CDW layer has the standard 3×3 kernel convolution and depth-wise convolution (DWConv2D) with 3×3 kernel to improve the channels and reduce the size, respectively. The outputs of the stem layer are 128×128×64, 64×64×128, 32×32×256, and 32×32×256, respectively, in the subsequent four blocks and the same as the outputs of the CDW layer. From Figure 3(b1-I–b4-I), in the feature extractor module, the scale of the feature map is decreased with multiples, while the channels are increased in dimension using multiples. The process of extracting high-dimensional information from Figure 3(b1-I–b4-I) involves calling three specific structures: the Conv1 group, the DWConv layer, and the AT layer. We describe the call relationship in the feature extractor submodule as follows:

(2) $$S_{i}=a\cdot CGroup+b\cdot DWLayer+c\cdot ATLayer$$

where the output $S_{i}$ in Equation (2) is the expansion variable in Equation (1) and denotes the feature extraction result of the stem layer. To describe the number of calls to the three structures at different stem layers, we use three coefficients: a, b, and c. Taking Figure 3(b1-I) as an example, the coefficients a, b, and c are each equal to 3, 1, and 1. In the same way, a, b, and c have the values 3, 0, and 1. In fact, we did not design a fixed matrix of coefficients for the stem layer when designing the feature extractor module in SPA-Net. These coefficients were dynamically adjusted during the experiment, and we present the adjusted results in this paper.

In the stem layers of the SPA-Net blocks, we replace the max-pooling layer, which is used in VGG16, with a DWConv layer. The max-pooling layer could lead some crucial information being dropped, but the problem can be efficiently avoided using convolution. The DWConv layer consists of DWConv2D and BN-activation, where the 3×3 filter kernel is responsible for one channel, and is only convolved by one kernel; therefore, the number of feature map channels generated in this process is exactly the same as the number of input channels. In addition, compared with standard convolution operations, DWConv2D has a lower parameter number and operation cost to change the size of feature map, Moreover, BN-Activation can help the process of stochastic gradient descent by smoothing and hiding the layer input distribution and alleviate the negative impact of weight updating of the stochastic gradient descent on subsequent layers. However, a DWConv layer is not applied in SPA-Net Block IV, to ensure the resolution of the feature map. Therefore, the DWConv2D in DWConvLayer can be defined as

(3) $$D_{\left(h,w,c\right)}=\sum W_{\left(3,3,c\right)}\cdot x_{\left(h/s,w/s,c\right)}$$

where *h*, *w*, and *c* represent the height, width, and channel of the input feature map *x*. We selected a fixed size 3×3 filter kernel for DWConv2D in our experiments, so the superscript of the kernel *W* was fixed to 3. In addition, we eliminated the effect of the size of the kernel using control of padding. Thus, the size of the DWConv2D output feature map is mainly determined by *s*, which determines whether the size of the input and output feature maps are *h* and *w* or halved, respectively.

**Figure 4:**
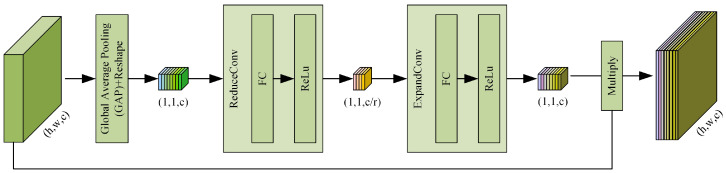
The detailed steps of the AT Layer.

Moreover, an attention (AT) layer follows the DWConv Layer. The DWConv layer and AT layer improve the efficiency and accuracy of visual information processing, which not only aims to speed up the network training but also makes the grid sparse, to reduce overfitting. Subsequently, we used an AT Layer. As shown in Figure 4, the AT Layer is composed of global average pooling (GAP), reshape, ReduceConv, and ExpandConv. The global compressed feature quantity of the current feature map is obtained by performing GAP on the feature map. The GAP method calculates the mean value of all pixels in each feature map, and its operation is as follows:

(4) $$D_{\left(1,1,c\right)}=\frac{1}{\left|R\right|}\sum D_{\left(h,w,c\right)}$$

where $D_{\left(1,1,c\right)}$ represents the output of GAP, $D_{\left(h,w,c\right)}$ is the output of DWConv Layer, and *R* is the number of pixels contained in a feature map in a single channel, with R=h*w. Weights for each channel in the feature map are obtained using the congestion structure for both layers. The weighted feature map is used as the input for the next layer, and 64 feature maps output 64 data points in Equation (4). These data-points have a 1×64 vector and reshape it into 1×1×64 features in Reshape, before ReduceConv and ExpandConv. The Fully Connected (FC)-ReLU forms ReduceConv and FC-Sigmoid forms of the ExpandConv operation, respectively, refer to squeeze and excitation, which generate a 1×1×16 feature map and 1×1×64 feature map, separately.Afterwards, 64 scalars between 0 and 1 are obtained as the weights of the channel, each output of the channel in the DWConv layer is weighted using the corresponding weight, each element of the corresponding channel is multiplied by the respective weight, and the new weighted feature is obtained. This operation can be summarized as a multiplication of the different channel feature maps using the global characteristics in Equation (5).

(5) $$DA_{\left(h,w,c\right)}=D_{\left(h,w,c\right)}\cdot D_{\left(1,1,c\right)}$$

As for the DWConv Layer in Stem Layer II, Stem Layer III and Stem Layer IV, it is the same as the DWConv Layer in Stem Layer I, to capture more useful information than the Max-pooling layer and reduce the number of parameters and computing cost more efficiently than standard convolution. In the AT Layer, for the first squeeze operation, we can obtain the global characteristics for the channel level. Then, we use the global characteristics control to learn the relationship between the various channels. We can obtain the weights for the different channels and multiply each channel by its corresponding weight. In essence, this AT layer performs attention operations on the channel dimension. This attention mechanism enables the model to pay more attention to channel features with the largest amount of information, while suppressing the unimportant channel features. Moreover, in all AT Layers in Stem Layers I-IV, the *r* in Figure 4 is 4, so the number of nodes in the first FC layer is 1/4 of the characteristic matrix channels of the input layer, and the number of nodes in the second FC layer is the same as that of the characteristic matrix channels of the input layer. After a series operation, the result of the residual branch is obtained, with 128×128×64. GAP is used to obtain the weights of each feature map channel, the Reshape step reshapes points to a tensor, ReduceConv reduces the channels to 1/4, and ExpandConv increases the channels to be same as the channels of feature map before the AT Layer.

### 3.3. Feature Matching Module

**Figure 5:**
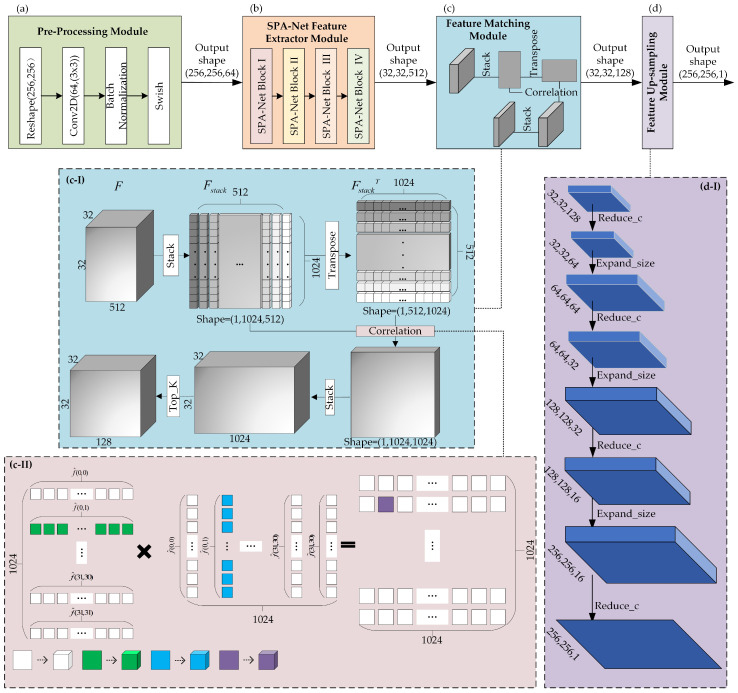
The structure of the feature matching module and feature upsampling module. (**a**–**d**) The four Modules of SPA-Net. (**c-I**) The global process of feature matching module. (**c-II**) The details of correlation in the feature matching module. (**d-I**) The intermediate outputs of the feature upsampling module, where Reduce_c is the convolution layer to reduce the channels of feature map, and Expand_size is used to upsample the feature map.

**Figure 6:**
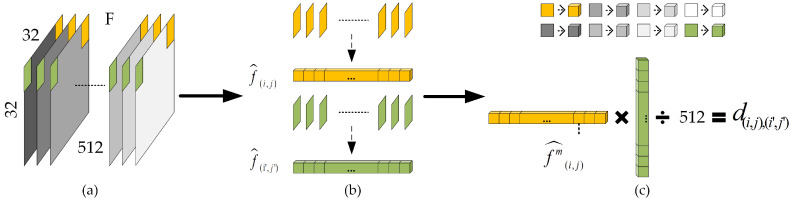
Illustration of the variation within the feature map, where each square represents a 1×1×1 cube: (**a**) is the extracted feature map F, (**b**) represents output of each patch and is of 1×1×512, and (**c**) shows the procedure for calculating the similarity between patches.

After extracting features with the SPA-Net feature extractor module, inspired by [18,20], a feature matching module is applied to match the characteristic representation and locate the tampered area by addressing the high-dimensional information. Figure 5 shows the feature matching module and feature upsampling module. The global structure of the feature matching module is shown in Figure 5(c-I). To begin with, we stack the *F* into $F_{stack}$ with shapes of 1×1024×512, which can be regarded as 512 patches with 1×1024, and ${\left(F_{\left(stack\right)}\right)}^{T}$ is a high-dimensional transpose matrix. To match the features, we need to calculate the correlation between each patch. The correlation distance is an efficient indicator to identify the inherent connections between each patch, so we use Equation (6) to calculate the correlation distance between $f_{\left(i,j\right)}$ and the sub-element $f_{\left(i^{\prime },j^{\prime }\right)}$ belonging to arbitrary coordinates $f_{\left(i,j\right)}$, as shown in Figure 6c.

(6) $$d_{\left(i,j\right),\left(i^{\prime },j^{\prime }\right)}={\hat{f}}_{\left(i,j\right)}\left({\hat{f}}_{\left(i^{\prime },j^{\prime }\right)}\right)/512$$

where (i,j) represents the index of patches in $F_{stack}$ and $\left(i^{\prime },j^{\prime }\right)$ represents the index of patches in ${\left(F_{\left(stack\right)}\right)}^{T}$, $i=\left\{0,..,31\right\}$, $j=\left\{0,..,31\right\}$ and the range of $\left(i^{\prime },j^{\prime }\right)$ is the same as (i,j). The $f_{\left(i,j\right)}$ and $f_{\left(i^{\prime },j^{\prime }\right)}$ are not a match when $d_{\left(i,j\right),\left(i^{\prime },j^{\prime }\right)}=0$. ${\hat{f}}_{\left(i,j\right)}$ and ${\hat{f}}_{\left(i^{\prime },j^{\prime }\right)}$ are standard deviations of $f_{\left(i,j\right)}$ and $f_{\left(i^{\prime },j^{\prime }\right)}$, which are shown in Figure 6b, and calculated using

(7) $${\hat{f^{m}}}_{\left(i,j\right)}=\frac{f_{\left(i,j\right)}^{m}-\mu f_{\left(i,j\right)}}{\sigma^{2}f_{\left(i,j\right)}}$$

where $\mu f_{\left(i,j\right)}$ is the mean of $f_{\left(i,j\right)}$, and $\sigma^{2}f_{\left(i,j\right)}$ is the variance of $f_{\left(i,j\right)}$. These are defined as

(8) $$f_{\left(i,j\right)}=\frac{1}{512}\sum_{a=1}^{512}f_{\left(i,j\right)}^{m}$$

(9) $$\sigma^{2}f_{\left(i,j\right)}=\frac{1}{512}\sum_{a=1}^{512}{\left(f_{\left(i,j\right)}^{m}-\mu f_{\left(i,j\right)}\right)}^{2}$$

Consequently, the correlation coefficient of $f_{\left(i,j\right)}$ with total calculate scores is denoted as $L_{\left(i,j\right)}$, $L_{\left(i,j\right)}=\left\{d_{\left(i,j),(0,0\right)},\ldots ,d_{\left(i,j\right),\left(i^{\prime },j^{\prime }\right)},\ldots ,d_{\left(i,j),(31,31\right)}\right\}$. Figure 5(c-II) describes the correlation step between every pair of patches in detail. Each patch generates a $L_{\left(i,j\right)}$ tensor of 1×1024 through a correlation calculation. Therefore, the final algorithm results in a correlation coefficient matrix of 32×32×1024. The example marked in Figure 5(c-II) shows the result of correlation calculation in the green part, blue part, and purple part. In order to better eliminate errors and over-fitting, we refine the output. Specifically, each $L_{\left(i,j\right)}$ is sorted from highest to lowest, and we select the top *K* patches that best match it, except itself. In SPA-Net, we select K=128, so we obtain a 32×32×128 correlation coefficient tensor after the feature matching module.

### 3.4. Feature Upsampling Module

The previous SPA-Net feature extraction module and feature matching module are used to analyze the local pixels of the image, so as to obtain high-order feature information; in this way, we can obtain the region of interest and general location information. In addition, due to the difference in size and channels between $\rho_{top}$ with 32×32×512 and input image with 256×256×3, we designed a feature upsampling module to recover the shape of $\rho_{top}$, in order to facilitate more intuitive observation of the results of our methods. Some interpolation methods do not learn parameters; for a deep learning architecture, these interpolation methods could affect the updating of parameters and thus reduce the accuracy of the architecture. Therefore, we chose transpose convolution in the feature upsampling module. Transpose convolution can, not only change the size and dimensions of the feature map, but also learn parameters, just like convolution. The change in the output shape is shown in Figure 5(d-I), where Reduce_c is a transpose convolution layer that has a kernel with 1×1 and the stride is 1, to reduce the channels of the feature map; and Expand_size is a transpose convolution layer, which has a kernel with the size of 3×3 and a stride of 2 to upsample the size.

## 4. Experiments and Discussions

In this section, we implemented the proposed SPA-Net model in the Tensorflow platform with Anaconda and Jupyter, and a GPU with Tesla V100-SXM3-32GB. As mentioned above, the existing pretrained DNN models are not suitable for our purposes, so we needed to train the model ourselves. In DNN training, the learning rate is a very important hyperparameter, because too large a learning rate cannot converge to the optimal solution and oscillates on both sides of the optimal solution, while too small a learning rate will cause the learning speed to be very slow in reducing the optimization speed. Therefore, we set the initial learning rate to 0.0001 at the beginning of training, for the purpose of obtaining a better solution quickly. Then, if the validation loss with the number of epochs reached 20, the learning rate would be halved, which made the model more stable during the training. The inputs of this model were the same size of 256×256 images.

To evaluate the performance of the proposed SPA-Net model, we calculated the metrics *Precision*, *Recall*, and *F1* [30]. *Precision* indicates the proportion of correctly detected forgery images/pixels of all detected images or pixels; *Recall* indicates the proportion of correctly detected forgery images or pixels of all forgery images or pixels; and *F1* is the combination of *Precision* and *Recall*. *Precision*, *Recall*, and *F1* are defined as:

(10) $$Presicion=\frac{TP}{\left(TP+FP\right)}$$

(11) $$Recall=\frac{TP}{\left(TP+FN\right)}$$

(12) $$F1=\frac{2\times (Precision\times Recall)}{\left(Precision+Recall\right)}$$

where *TP* is the true positives, the number of forged pixels predicted as forged; *FP* is false positives, the number of genuine pixels predicted as forged; and *FN* is False Negatives, the number of forged pixels predicting as genuine.

### 4.1. Datasets

#### 4.1.1. Training Dataset

**Figure 7:**
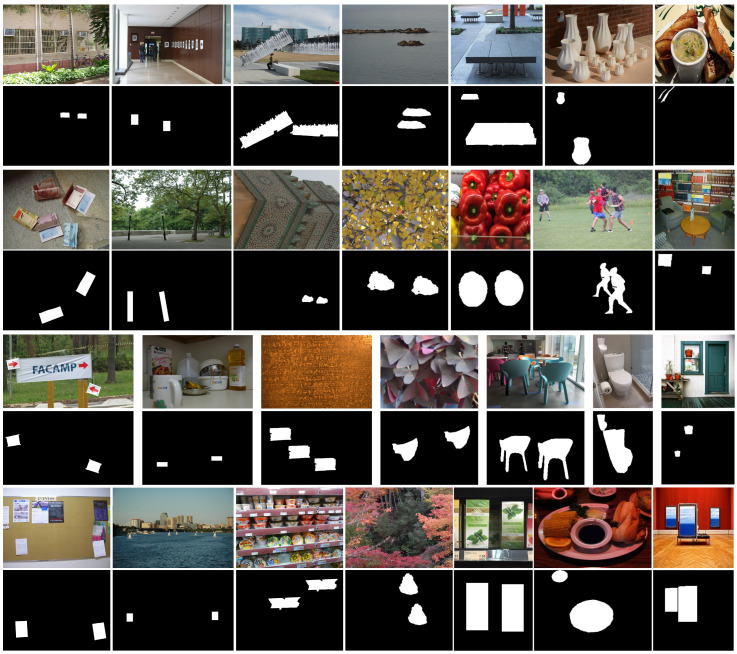
Copy-move images and their corresponding masks on the training dataset. Columns 1–7 are from CMH [31], MICC-F220 [32], MICC-F600 [33], GRIP [34], Coverage [35], USCISI-CMFD [18], and SPANet-CMFD.

None of the existing datasets for CMFD provide a sufficient number of images and corresponding ground truth masks for training. Since we did not use a pretraining model, we needed many images containing a copy-move forgery area to train our proposed network. Therefore, in this paper, we built a SPANet-CMFD dataset. With original images from SUN [24] and COCO [25], we selected the source area using Labelme manually for each image. During the generation, we selected 550 images from SUN [24] and COCO [25], and applied attacks including rotation with rotation angles of 5°, 20°, 45°, 60°, 90°, and 180°, and scaling with scaling degree of 30%, 50%, 100%, 110%, 120%, and 150%, to the copied regions. In addition, when non-electronic tampered images are used with our detector, they are generally sent to our detector through sensors, such as scanners and cameras. In the process of sampling and transmission, visual sensors will generally have some impact on image quality, such as extra blur and noise. Therefore, in order to increase the robustness of our network, during the production of the training sets, different kinds of postprocessing were applied after rotation and scaling, including contrast adjustments (CA) with (0.01, 0.95), and (0.01, 0.9), (0.01, 0.8); image blurring (IB) with 3 × 3, 5 × 5; Gaussian noise addition (NA) with degrees from 0.2 to 1; and brightness changing (BC) with factors of (0.01, 0.95), (0.01, 0.09), and (0.01, 0.8). The number of forgery images in the SPANet-CMFD dataset was 550 × 12 × 11 = 72,600. We selected about 50,000 useful images. In addition to the SPANet-CMFD dataset, we also used 50,000 images from [18], and some other public copy-move datasets for training, including CMH [31], MICC-F220 [32], MICC-F600 [33], GRIP [34], and Coverage [35], and we also carried out pos-processing for these datasets, so we obtained 558 × 11 = 6138 copy-move forgery samples. Figure 7 shows several images and their corresponding masks on training dataset.

CMH [31]: This consists of four CMH sub-datasets, with a total of 108 copy-move tampered images, which involve attacks with rotation and scaling transformations.

MICC-F220 [32]: There are 110 bases and 110 tampered images, with sizes ranging from 722 × 480 to 800 × 600. However, the dataset does not provide a ground truth for tampered images, we used Labelme to make a ground-truth for them.

MICC-F600 [33]: This consists of 160 tampered images and 440 original images, with a resolution ranging from 800 × 533 to 3888 × 2592.

GRIP [34]: There are 80 basic images and 80 corresponding tampered images with a size of 768×1024, and the dataset provides a corresponding ground truth. Most of the duplicated pieces in the image are smooth.

Coverage [35]: This dataset has 100 basic images and corresponding tampered images with an average size of 400 × 486.

USCISI-CMFD [18]: We selected 50,000 copy images and their ground-truths randomly.

#### 4.1.2. Testing Dataset

To facilitate comparison with the existing state-of-the-art CMFD methods [9,17,18,21,31,36,37,38], we used two public datasets, CASIA [39] and CoMoFoD [40], as our testing dataset. CASIA [39] contains 5123 splicing and copy-move forged images, involving attacks with rotation and scaling, and we imitated [18,21] and selected 1266 copy-move forged images for the test. CoMoFoD [40] contains 200 basic images and 4800 copy-move forged images generated by applying various post-processing approaches, including JPEG compression, CA, NA, IB, BC, and color reduction (CR); and the setting details of these attacks are listed in Table 1.

### 4.2. Performance and Comparison with the CASIA Dataset

Table 2 shows the comparison results of our method with the other state-of-the-art methods [9,17,18,21,36,37], on the CASIA dataset. The compared works [9,36,37] were traditional methods, where [9,36] are block-based methods, and [37] is a keypoint-based method. Where [18,21] and our method are DNN-based. It can be seen from Table 2 that [9,36] performed significantly worse than the keypoint-based method [37], because most of the images in the CASIA dataset were processed using large-scaling, while the block-based methods, [9,36] were not robust against this kind of attack. On the contrary, the key-point-based approaches were robust against large-scale distortions, but they were not good at dealing with smooth areas. For the deep learning methods, except [17] which depends on obviously tampered edges, such as splicing feature information, the mehod of reference [18,21] and ours obtain the best *F1*, because the deep learning methods could resolve the disadvantages of the traditional methods. Overall, our approach learned useful rich feature information with a span-partial structure with an attention mechanism and had outstanding performance, and the training dataset of SPA-Net included much rotation and scaling of images.

**Figure 8:**
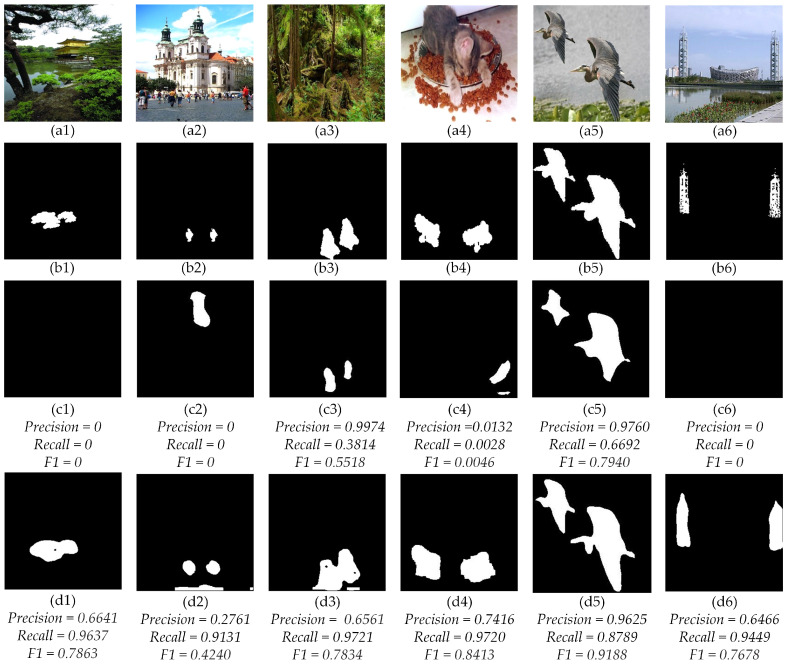
Comparison of BusterNet [18] and the proposed SPA-Net on the CASIA dataset. (**a1**–**a6**) The copy-move images, (**b1**–**b6**) The groud-truth of copy-move images, (**c1**–**c6**) The results of BusterNet [18], (**d1**–**d6**) The results of SPA-Net.

Figure 8 shows a visualization comparison between the state-of-the-art work BusterNet [18] and our proposed SPA-Net. The 1st and 2nd rows show the forgery images and the corresponding ground-truth. The 3rd row sshows the detection results of BusterNet [18], with the {*Precision*, *Recall*, *F1*} calculated as {0, 0, 0}, {0, 0, 0}, {0.9974, 0.3814, 0.5518}, {0.0132, 0.0028, 0.0046}, {0.9760, 0.6692, 0.7940}, and {0, 0, 0}, respectively. The 4th row shows the corresponding results of the proposed SPA-Net, with the {*Precision*, *Recall*, *F1*} calculated as {0.6641, 0.9637, 0.7863}, {0.2761, 0.9131, 0.4240, {0.6561, 0.9721, 0.7834}, {0.7416, 0.9720, 0.8413}, {0.9265, 0.8789, 0.9188}, and {0.6466, 0.9449, 0.7678}, respectively. The comparison results demonstrated the significantly better performance of the proposed SPA-Net. The results of (a1) and (a4) showed that our method was better at scaling and rotation than BusterNet [18], the results of (a2) showed that BusterNet [18] was poor at dealing with smaller areas.

### 4.3. Performance Comparison on the Comofod Dataset

Table 3 shows the pixel-level results of [9,17,18,31,38] and ours, where [9,31,38] are traditional methods, and reference [17,18] and ours are DNN-based methods. It is worth mentioning that compared with deep learning-based methods, traditional methods lack universality and stability. In addition, our method outperformed Deep-L [17] and BusterNet [18], which are both deep-learning-based methods, and it can also be seen that the performance of the proposed SPA-Net was outstanding under most attacks.

**Figure 9:**
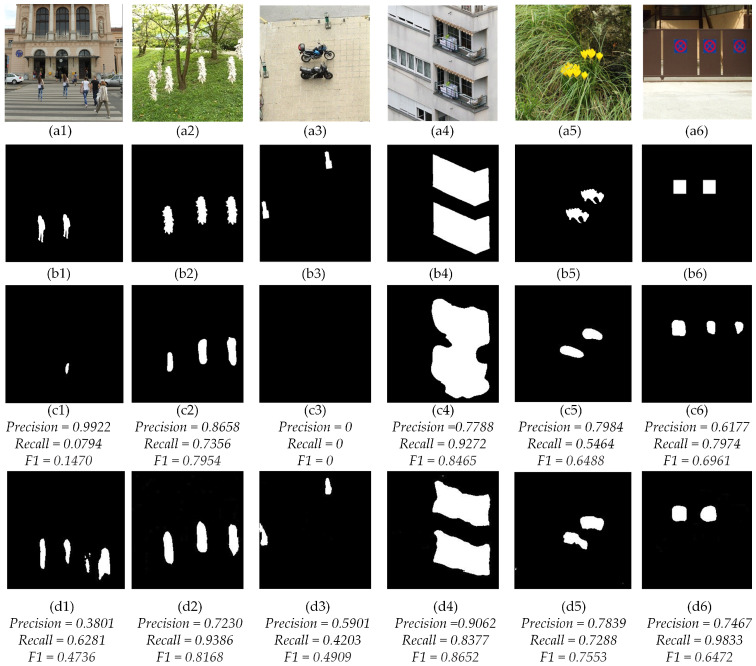
Comparison of BusterNet [18] and proposed SPA-Net on CoMoFoD Dataset. (**a1**–**a6**) The copy-move images, (**b1**–**b6**) The groud-truth of copy-move images, (**c1**–**c6**) The results of BusterNet [18], (**d1**–**d6**) The results of SPA-Net.

Figure 9 shows the visualization comparison between the state-of-the-art work BusterNet [18] and our proposed SPA-Net. The 1st and 2nd rows show the forgery images and the corresponding ground-truth. The 3rd row shows the detection results of BusterNet [18], with the {*Precision*, *Recall*, *F1*} calculated as {0.9922, 0.0794, 0.1470}, {0.8658, 0.7456, 0.7954}, {0, 0, 0}, {0.7788, 0.9272, 0.8456}, {0.7984, 0.5464, 0.6488} and {0.6177, 0.7974, 0.6961}, respectively. The 4th row shows the corresponding results of the proposed SPA-Net, with the {*Precision*, *Recall*, *F1*} calculated as {0.3801, 0.6281, 0.4736}, {0.7230, 0.9386, 0.8168}, {0.5901, 0.4203, 0.4909}, {0.9062, 0.8377, 0.8652}, {0.7839, 0.7288, 0.7533}, and {0.7467, 0.9833, 0.6472}, respectively. The comparison results demonstrate the significantly better performance of the proposed SPA-Net. In the results of (a1) and (a4), BusterNet [18] can not detect the small copy-move areas, but ours methods can, in (d1) we detect some genuine pixels as copy-move areas, so the *Recall* is higher. our method outperforms BusterNet [18] in *Precision*, *Recall* and *F1* in the results of (a6), because BusterNet [18] detect other similiary areas as the copy-move areas.

## 5. Conclusions

In this paper, we proposed an end-to-end neural network SPA-Net for CMFD. In the SPA-Net Feature Extractor Module, the gradients from left branch of the stem layer and right branch of the Conv_BN_Leaky Relu (CBLR) are integrated without duplicate feature information, we also pay sufficient attention to the correlation between high-dimensional feature channels, and use the corresponding enhanced Attention Mechanism to find the potential characteristic representation. The Feature Matching Module can be more flexible adapted to the need for high-dimensional matching in deep learning. As well, the Feature Up-Sampling Module is easier to be embedded in SPA-Net. Compared with the traditional linear interpolation, the transposed convolution in Up-Sampling Module is learnable. Moreover, we create the SPANet-CMFD dataset by applying various processing on the benchmark images from SUN and COCO datasets. Next, we use the existing copy move forgery datasets, CMH, MICC-F220, MICC-F600, GRIP, Coverage, and USCISI-CMFD, together with our generated SPANet-CMFD dataset as the training set to train our model. Therefore, the inclusiveness of copy move tampering part of the network is improved. Compared with other CMFD methods, the proposed SPA-Net achieves good results in both CASIA dataset and CoMoFoD dataset. According to the evaluation results of the test datasets, the generalization ability of SPA-Net is better than most traditional and deep learning methods, but the ability of SPA-Net to detect duplicate areas of the image still needs to be improved, especially improve the detection ability of smaller copy-move areas. Therefore, in the future research, we will improve from two aspects. Firstly, we will combine SPA-Net with traditional methods to improve network generalization ability and eliminate mismatched areas. Next, on the basis of the network structure using deep learning, we should also pay attention to the popular image processing research fields, such as image segmentation, because CMFD and image segmentation have the same characteristic of detecting the location, so they can be combined in the feature extraction or result optimization stage, and it will have a certain effect on the improvement of network performance. Additionally, in terms of application, SPA-Net can be regarded as a sub-stage to distinguish the source and target areas in the future, it can also be combined with smart visual sensors to automatically analyze and locate copy-move forgery of images. Moreover, the generation of SPANet-CMFD can also be made into other datasets with simple processing, such as splicing and removal, as well as it can be applied to any field related to image tampering, such as remote sensing and deepfake.

## Figures and Tables

**Table 1:** The setting details of each attack with different levels, with the CoMoFoD database.

Attacks	Level Settings
Brightness Changing (BC)	Brightness ranges: [(0.01, 0.95), (0.01, 0.9), (0.01, 0.8)]
Contrast Adjustment (CA)	Adjustment ranges: [(0.01, 0.95), (0.01, 0.9), (0.01, 0.8)]
Color Reduction (CR)	Intensity levels per each color channel: [32, 64, 128]
Image Blurring (IB)	Average filters: [3 × 3, 5 × 5, 7 × 7]
JPEG Compression (JC)	Compression factors: [20, 30, 40, 50, 60, 70, 80, 90, 100]
Noise Adding (NA)	Variance: [0.009, 0.005, 0.0005]

**Table 2:** Comparison of performance on the CASIA dataset.

Methods	*Precision*	*Recall*	*F1*
Zernike [9]	0.227	0.134	0.164
PCET [36]	0.311	0.294	0.302
Over-Seg [37]	0.430	0.367	0.396
Deep-L [17]	0.240	0.138	0.175
BusterNet [18]	0.557	0.438	0.456
AR-Net [21]	0.583	0.373	0.455
Ours	0.534	0.431	0.465

**Table 3:** Comparison of the CMFD results on the CoMoFoD dataset at pixel level.

Methods	*Precision*	*Recall*	*F1*
Zernike [9]	0.336	0.325	0.330
Deeper [31]	0.067	0.066	0.067
Seg [38]	0.089	0.088	0.089
Deep-L [17]	0.316	0.296	0.306
BusterNet [18]	0.404	0.333	0.365
Ours	0.413	0.360	0.385

## Data Availability

Publicly available datasets were analyzed in this study. This data can be found here: CMH: http://dx.doi.org/10.6084/m9.figshare.978736, accessed on 10 January 2021. MICC-F220: http://www.micc.unifi.it/downloads/MICC-F220.zip, accessed on 12 January 2021. MICC-F600: http://www.micc.unifi.it/downloads/MICC-F600.zip, accessed on 12 January 2021. GRIP: http://www.grip.unina.it/, accessed on 16 March 2021. Coverage: https://github.com/wenbihan/coverage, accessed on 1 January 2021. USCISI-CMFD: https://github.com/isi-vista/BusterNet/tree/master/Data/USCISI-CMFD-Small, accessed on 2 February 2021. CASIA: https://github.com/isi-vista/BusterNet/tree/master/Data/CASIA-CMFD, accessed on 2 February 2021. CoMoFoD: https://www.vcl.fer.hr/comofod/, accessed on 18 June 2021.
